# Self-Reported Real-World Safety and Reactogenicity of COVID-19 Vaccines: A Vaccine Recipient Survey

**DOI:** 10.3390/life11030249

**Published:** 2021-03-17

**Authors:** Alexander G. Mathioudakis, Murad Ghrew, Andrew Ustianowski, Shazaad Ahmad, Ray Borrow, Lida Pieretta Papavasileiou, Dimitrios Petrakis, Nawar Diar Bakerly

**Affiliations:** 1 Division of Infection, Immunity and Respiratory Medicine, School of Biological Sciences, The University of Manchester, Manchester M23 9LT, UK; alexander.mathioudakis@manchester.ac.uk; 2 North West Lung Centre, Wythenshawe Hospital, Manchester University NHS Foundation Trust, Manchester Academic Health Science Centre, Manchester M23 9LT, UK; 3 Department of Respiratory Medicine, Salford Royal Hospital NHS Foundation Trust, Manchester M6 8HD, UK; muradghrew@gmail.com; 4 Department of Intensive Care Medicine, Salford Royal Hospital NHS Foundation Trust, Manchester M6 8HD, UK; 5 Faculty of Biology, Medicine & Health, School of Biological Sciences, The University of Manchester, Manchester M13 9PL, UK; Andrew.Ustianowski@pat.nhs.uk; 6 Regional Infectious Diseases Unit, North Manchester General Hospital, Manchester M8 5RB, UK; 7 Department of Virology, Manchester Medical Microbiology Partnership, Manchester University NHS Foundation Trust, Manchester M13 9WL, UK; Shazaad.Ahmad@mft.nhs.uk; 8 Vaccine Evaluation Unit, Public Health England, Manchester Royal Infirmary, Manchester University NHS Foundation Trust, Manchester Academic Health Science Centre, Manchester M13 9WL, UK; Ray.Borrow@phe.gov.uk; 9 Department of Cardiology, Hygeia Hospital, 15123 Athens, Greece; lidapieretta@hotmail.com; 10 Allergy Clinic, Petrakis Allergy Care, 55133 Thessaloniki, Greece; petrakis.d@gmail.com; 11 School of Healthcare Sciences, Manchester Metropolitan University, Manchester M15 6BH, UK

**Keywords:** Coronavirus Disease 2019, COVID-19, COVID-19 vaccine, safety, reactogenicity, tolerability, adverse events

## Abstract

An online survey was conducted to compare the safety, tolerability and reactogenicity of available COVID-19 vaccines in different recipient groups. This survey was launched in February 2021 and ran for 11 days. Recipients of a first COVID-19 vaccine dose ≥7 days prior to survey completion were eligible. The incidence and severity of vaccination side effects were assessed. The survey was completed by 2002 respondents of whom 26.6% had a prior COVID-19 infection. A prior COVID-19 infection was associated with an increased risk of any side effect (risk ratio 1.08, 95% confidence intervals (1.05–1.11)), fever (2.24 (1.86–2.70)), breathlessness (2.05 (1.28–3.29)), flu-like illness (1.78 (1.51–2.10)), fatigue (1.34 (1.20–1.49)) and local reactions (1.10 (1.06–1.15)). It was also associated with an increased risk of severe side effects leading to hospital care (1.56 (1.14–2.12)). While mRNA vaccines were associated with a higher incidence of any side effect (1.06 (1.01–1.11)) compared with viral vector-based vaccines, these were generally milder (*p* < 0.001), mostly local reactions. Importantly, mRNA vaccine recipients reported a considerably lower incidence of systemic reactions (RR < 0.6) including anaphylaxis, swelling, flu-like illness, breathlessness and fatigue and of side effects requiring hospital care (0.42 (0.31–0.58)). Our study confirms the findings of recent randomised controlled trials (RCTs) demonstrating that COVID-19 vaccines are generally safe with limited severe side effects. For the first time, our study links prior COVID-19 illness with an increased incidence of vaccination side effects and demonstrates that mRNA vaccines cause milder, less frequent systemic side effects but more local reactions.

## 1. Introduction

Coronavirus Disease 2019 (COVID-19) rapidly became a leading cause of death and short and long-term morbidity among people over the age of 45 [1,2], posing an unprecedented burden to healthcare systems with worldwide economic consequences and prolonged lockdowns [3]. Vaccines currently being rolled out are anticipated to significantly modify these trends. While their effectiveness and safety have been proven in recent studies [4,5,6], data in specific groups remain lacking. Generally, people with a previous history of COVID-19 in whom vaccination is currently advised [7] were excluded from the clinical trials [4,5,6]. Whilst it is accepted that prior infection with COVID-19 induces a natural immunity potentially lasting for at least six months [8], it is unknown if previous infection may be associated with a greater number of vaccination side effects. Moreover, the safety and reactogenicity of the different types of vaccines (mRNA or viral vector-based) have not been compared head-to-head. This anonymized online survey was conducted to compare the safety profiles of available COVID-19 vaccines and evaluate their side effects in different groups of vaccine recipients.

## 2. Materials and Methods

This online survey, developed in plain English language and piloted by experts and lay people, captured basic epidemiological data, details on COVID-19 exposure, vaccination history and the incidence and severity of the respective side effects (Appendix A: Table A1). More specifically, we enquired about the following symptoms: localized reactions (pain, swelling, tenderness, redness, itching or other), fever, skin rash, shortness of breath, tingling in the mouth, face, body/extremities, swelling in the face or mouth, generalized swelling, anaphylaxis (severe allergic reaction with face swelling and breathlessness), tiredness or fatigue, flu-like illness or any other side effects. It was launched via Google Forms on 3 February 2021 for 11 days and was shared within the institutions of the investigators through professional contacts and social media. The only inclusion criterion was the receipt of the first dose of any COVID-19 vaccine at least seven days prior to survey completion.

The main objectives were to evaluate the differences in the incidence and severity of vaccination side effects among (i) people with versus without previously reported COVID-19 infection and (ii) those who received different vaccine types. Moreover, we explored the differences in self-reported side effects between the first and second vaccine dose among different ethnicities and among those with different preconceptions toward the vaccine. Finally, we explored the impact of the interval between COVID-19 exposure and vaccination and the incidence of side effects.

For our main analysis, a positive COVID-19 history was considered in cases of (a) a self-reported history of symptoms consistent with COVID-19 disease provided that COVID-19 was not excluded by a negative PCR test, (b) a positive COVID-19 PCR test or (c) a positive COVID-19 antigen test. In a sensitivity analysis, a COVID-19 infection was only considered valid if it was confirmed by PCR or antigen testing while patients with an uncertain exposure (clinical history not confirmed by laboratory testing) were excluded.

Between group differences were assessed using chi-squared and Mann–Whitney U tests for dichotomous and continuous variables, respectively, after a Shapiro–Wilk test excluded the normal distribution of the latter. Between group differences in the incidence of side effects are presented as risk ratios (RR) with the respective 95% confidence intervals (CI). Predictors of the incidence and severity of side effects were evaluated in univariate followed by multivariate binomial logistic regression and cumulative link models for ordinal data, respectively. Age, gender, ethnicity, vaccine type, prophylactic analgesia or other medication use prior to vaccination, vaccine preconceptions and prior COVID-19 exposure were evaluated as potential confounding factors. Unless otherwise specified, the analyses were based on side effect profiles from the first dose of the vaccine.

Ethics approval was not necessary for this anonymized survey.

## 3. Results

**Figure A1:**
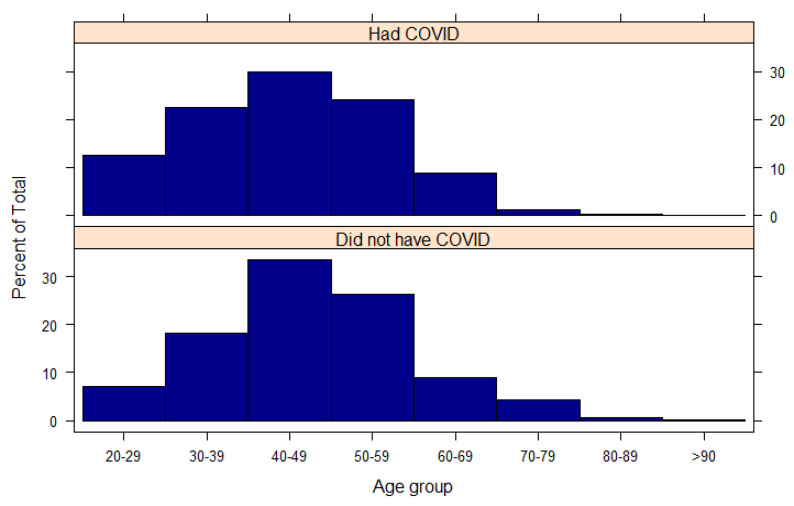
Age of the participants stratified by whether they had or did not have a previous COVID-19 infection.

**Figure A2:**
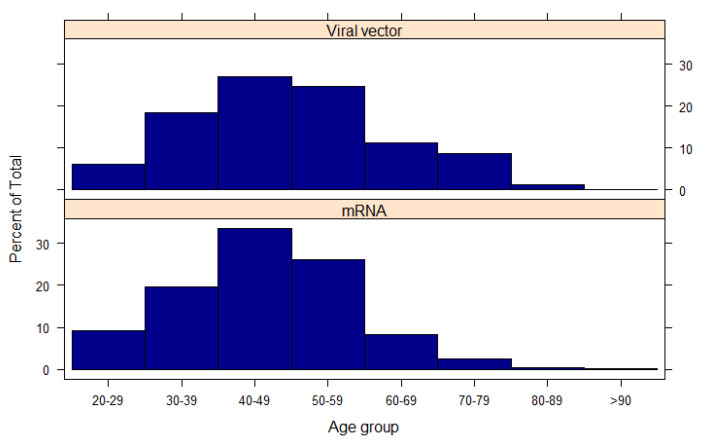
Age of the participants stratified by the type of vaccine they received.

Within 11 days, this online survey was completed by 2002 participants (Table A2, Figure A1), mostly health professionals of a working age (median: 45, interquartile range [IQR]: 35–50 years). A total of 532 (26.6%) had a history of a previous COVID-19 infection of whom 366 (68.8%) were confirmed by PCR (*n* = 273) and/or antigen testing (*n* = 162). A COVID-19 infection preceded the first vaccination dose by a median of 87 (IQR: 47–223) days. The majority of respondents were Caucasians (88.3%) mostly from the UK (78.6%) and Greece (16.6%). As anticipated, a prior history of a COVID-19 infection was more prevalent among frontline workers, health professionals and people from the UK where a very high incidence of COVID-19 was documented [9]. Moreover, recipients of a viral vector-based vaccine (mainly the AstraZeneca vaccine) were relatively older (Figure A2, *p* < 0.001) and were mostly based in the UK (89.7% compared with 76.4% of those that received viral mRNA vaccines, *p* < 0.001). Finally, doctors were more likely to have received an mRNA-based vaccine compared with the other groups (*p* < 0.001).

**Figure 1:**
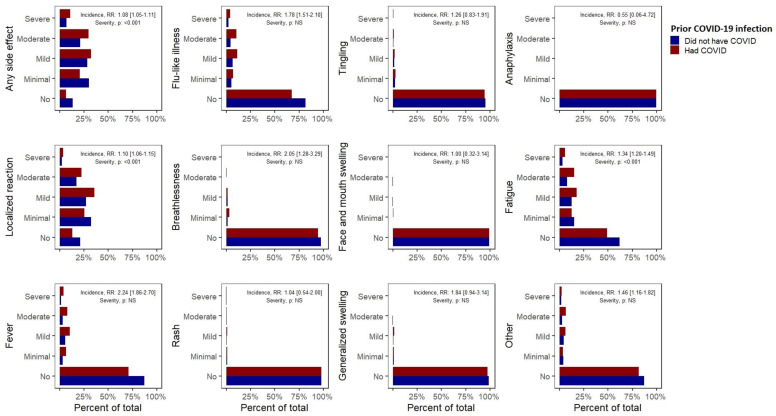
Incidence and severity of self-reported side effects after the first dose of the COVID-19 vaccine among participants who had or did not have a known prior COVID-19 infection. Risk ratios less than 1 favoured those that did not have a prior COVID-19 infection.

A prior COVID-19 infection was associated with an 8% increase in the risk of having any side effects after the first vaccine dose (RR 1.08, 95% CI (1.05–1.11), Table 1, Figure 1). We also observed a significantly increased risk of self-reported fever (2.24 (1.86–2.70)), breathlessness (2.05 (1.28–3.29)), flu-like illness (1.78 (1.51–2.10)), fatigue (1.34 (1.2–1.49)), local reactions (1.10 (1.06–1.15)) and “other” side effects (1.46 (1.16–1.82)). Among those experiencing side effects, a prior COVID-19 infection was associated with an increased severity of any side effect, local side effects or fatigue (*p* < 0.001). More importantly, a prior COVID-19 infection was associated with the risk of experiencing a severe side effect requiring hospital care (1.56 (1.14–2.12)). These observations remained significant in multivariate analyses and our sensitivity analysis (Table A3). A similar increase in the risk of any side effects following the second dose in those with a prior COVID-19 infection was also noted (1.08 (1.05–1.11)), although the lack of significant associations with specific side effects may have resulted from the limited sample included in this analysis.

**Figure 2:**
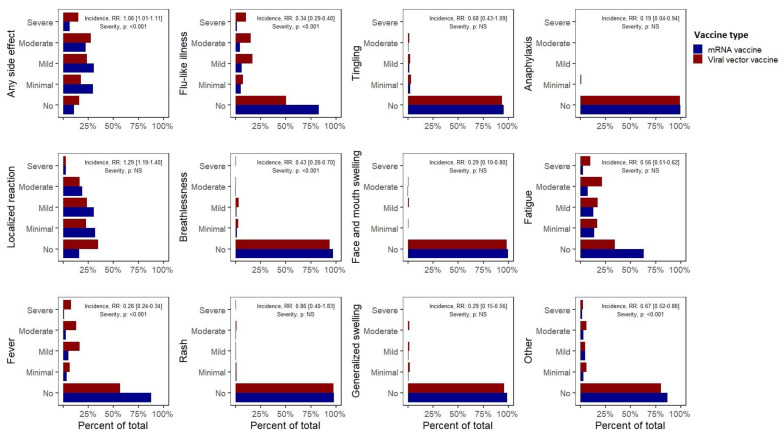
Incidence and severity of side effects after the first dose of (1) an mRNA or (2) a viral vector vaccine. Risk ratios less than 1 favoured the mRNA vaccine.

Furthermore, significant differences were observed between the side effect profiles of mRNA versus viral vector vaccines (predominantly Pfizer versus AstraZeneca, Table 2, Figure 2). Overall, the recipients of mRNA vaccines reported a higher incidence of any self-reported side effects (1.06 (1.01–1.11)), which were, however, of significantly milder severity compared with those who received viral vector vaccines. While mRNA vaccines were associated with an increased incidence of reported local reactions (1.29 (1.19–1.40)), they were associated with a considerably lower incidence of self-reported systemic side effects including anaphylaxis (0.19 (0.04–0.62)), fever (0.28 (0.24–0.34)), swelling in the face or mouth (0.29 (0.10–0.80)) or generalized swelling (0.29 (0.15–0.56)), flu-like illness (0.34 (0.29–0.40)), breathlessness (0.43 (0.26–0.70)), fatigue (0.56 (0.51–0.62)) or other side effects (0.67 (0.52–0.86)). These observations were corroborated by multivariate analyses. Most importantly, mRNA vaccines were associated with a significantly lower incidence of severe side effects (requiring hospital care, RR 0.42 (0.31–0.58)).

In general, the second dose of the vaccine was associated with a higher incidence of side effects (Table 3). More specifically, respondents reported experiencing more frequently any side effects (1.04 (1.01–1.07)), skin rash (2.25 (1.4–3.62)), fever (1.72 (1.46–2.02)), flu-like illness (1.67 (1.45–1.91)) and fatigue (1.40 (1.28–1.53)). In addition, a multivariate regression demonstrated that participants who had side effects after the first vaccine dose were at a significantly higher risk of having the same side effects after the second dose. Among those experiencing side effects, the severity did not significantly differ between the two doses. However, the likelihood of having a severe side effect requiring hospital care was significantly decreased (0.58 (0.38–0.88)).

Stratification by ethnicity revealed that white participants reported a lower incidence of fever (0.62 (0.48–0.79)) and flu-like illness (0.78 (0.62–0.97)) compared with the remaining participants (Table A4). Finally, those reporting a pre-vaccination concern about the safety of the vaccine reported more often tingling (2.23 (1.45–3.42)), breathlessness (1.73 (1.00–2.98)) and fatigue (1.17 (1.03–1.34)) (Table A5).

Multivariate analyses also revealed a strong negative association between age and the self-reporting of any side effect, local reactions, fever, flu-like illness, rash, tingling, generalized swelling and fatigue (*p* < 0.01). Finally, a history of allergy was associated with an increased incidence of self-reported breathlessness and rash (*p* < 0.01). However, as described in the previous paragraphs and tables, most of the associations observed in univariate analyses remained significant in multivariate analyses accounting for these and other potential confounding factors.

## 4. Discussion

People with a prior COVID-19 exposure were largely excluded from the vaccine trials [4,5,6] and, as a result, the safety and reactogenicity of the vaccines in this population have not been previously fully evaluated. For the first time, this study demonstrated a significant association between a prior COVID-19 infection and a significantly higher incidence and severity of self-reported side effects after a vaccination for COVID-19. Consistently, compared with the first dose of the vaccine, we found an increased incidence and severity of self-reported side effects after the second dose when recipients had been previously exposed to viral antigen, probably because they had already developed an immunity against the antigens. This was supported by recent studies demonstrating that seropositive individuals developed rapid immune responses with higher antibody titres after the first vaccination dose compared with those without a previous COVID-19 infection [10,11]. In view of the rapidly accumulating data demonstrating that COVID-19 survivors generally have an adequate natural immunity for at least six months, it may be appropriate to re-evaluate the recommendation for the immediate vaccination of this group. In the meantime, taking into account our findings as well as studies demonstrating higher antibody titres among individuals with a prior COVID-19 infection, it might be appropriate for a note to be included in the vaccine information sheets highlighting that these people are more likely to experience non-serious side effects.

Moreover, this is the first head-to-head real-world comparison of the self-reported safety of viral vector versus mRNA vaccines with the latter associated with a 58% decreased incidence of self-reported severe side effects requiring hospital care. While a greater number of recipients of mRNA vaccines reported at least one (any) side effect, the difference was predominantly driven by the frequent local reactions. The incidence of the systemic side effects evaluated (flu-like illness, pyrexia and fatigue), which are more burdensome to the recipients, was significantly reduced. The recipients of the viral vector-based vaccines were relatively older. However, differences in the incidence of adverse events were confirmed in multivariate analyses accounting for the age of the respondents as a covariate. Moreover, given that older people reported side effects less frequently, a potential bias due to age difference would be expected to favour viral vector-based vaccines. These findings may have an impact on vaccine choice and health policies. The cause of the observed imbalance between the safety profiles of mRNA-based versus viral-vector vaccines was unclear and should be evaluated in future studies.

The main strengths of our study included a large study population that better reflected real-life compared with the populations studied in the clinical trials, the availability of adequate details about the participants and the safety profiles of the vaccines and very limited missing data. The potential bias of respondents is the main limitation of any survey and as this survey was shared though social media, we were not able to estimate the non-response rate. However, the bias of respondents was more likely to affect the absolute incidence of side effects, which we did not evaluate here, rather than the relative incidence and severity across different groups of people. Potential recall bias should also be mentioned although all participants had been vaccinated within 10 weeks prior to completing the survey. As noted, most respondents were from the UK and Greece due to the ability of the investigators to establish contacts quickly to publicise this survey. The UK has also been successful in rolling out COVID-19 vaccines quickly leading to more of those invited being eligible to participate. It is not surprising that the Pfizer vaccine was the most delivered vaccine as it was the first vaccine to be licensed within the UK, with more individuals receiving it in total when the survey was circulated.

In conclusion, this extensive survey of over 2000 recipients of COVID-19 vaccines confirmed the findings of recent randomised controlled trials (RCTs) demonstrating that COVID-19 vaccines are generally safe with limited severe side effects. Moreover, it linked previous COVID-19 illnesses with an increased incidence of vaccination side effects. It also demonstrated that mRNA vaccines caused milder, less frequent systemic side effects but more local reactions. These findings will need to be validated in clinical studies, preferably randomized controlled trials including patients from multiple groups.

## Figures and Tables

**Table 1:** Differences in the incidence and severity of side effects after the first dose of the COVID-19 vaccine among participants who had or did not have a prior COVID-19 infection.

Side Effect	Incidence of Side Effects: Risk Ratio (95% CI)	Incidence of Side Effects: Multivariate Logistic Regression, Coefficient (*p*-Value)	Severity of Side Effects: Univariate Cumulative Risk Models (*p*-Value)	Severity of Side Effects: Multivariate Cumulative Risk Models (*p*-Value)
Any side effect	1.08 (1.05–1.11)	0.575 (0.004)	<0.001	<0.001
Localized reaction	1.10 (1.06–1.15)	0.45 (0.003)	<0.001	0.003
Fever	2.24 (1.86–2.70)	0.876 (<0.001)	NS	NS
Flu-like illness	1.78 (1.51–2.10)	0.658 (<0.001)	NS	NS
Shortness of breath	2.05 (1.28–3.29)	0.651 (0.011)	NS	NS
Skin rash	1.04 (0.54–2.00)	NS	NS	NS
Tingling	1.26 (0.83–1.91)	NS	NS	NS
Swelling	1.00 (0.32–3.14)	NS	NS	NS
Generalized swelling	1.84 (0.94–3.60)	NS	NS	NS
Anaphylaxis	0.55 (0.06–4.72)	NS	NS	NS
Fatigue or tiredness	1.34 (1.2–1.49)	0.418 (<0.001)	<0.001	<0.001
Other	1.46 (1.16–1.82)	0.349 (0.013)	NS	NS
		Worse outcomes associated with a prior COVID-19 infection		

**Table 2:** Differences in the incidence and severity of side effects among people who received an mRNA or a viral vector vaccine.

Side Effect	Incidence of Side Effects: Risk Ratio (95% CI)	Incidence of Side Effects: Multivariate Logistic Regression, Coefficient (*p*-Value)	Severity of Side Effects: Univariate Cumulative Risk Models (*p*-Value)	Severity of Side Effects: Multivariate Cumulative Risk Models (*p*-Value)
Any side effect	1.06 (1.01–1.11)	NS	<0.001	<0.001
Localized reaction	1.29 (1.19–1.40)	0.892 (<0.001)	NS	NS
Fever	0.28 (0.24–0.34)	−1.993 (<0.001)	<0.001	NS
Flu-like illness	0.34 (0.29–0.40)	−1.795 (<0.001)	<0.001	NS
Shortness of breath	0.43 (0.26–0.70)	−0.853 (0.002)	NS	NS
Skin rash	0.86 (0.40–1.83)	NS	NS	NS
Tingling	0.68 (0.43–1.09)	NS	NS	NS
Swelling	0.29 (0.10–0.80)	−1.326 (0.015)	NS	NS
Generalized swelling	0.29 (0.15–0.56)	−1.423 (<0.001)	NS	NS
Anaphylaxis	0.19 (0.04–0.94)	−1.890 (0.024)	NS	NS
Fatigue or tiredness	0.56 (0.51–0.62)	−1.331 (<0.001)	<0.001	NS
Other	0.67 (0.52–0.86)	−0.471 (0.004)	NS	NS
		mRNA vaccines superiority		
		Viral vector vaccines superiority		

**Table 3:** Differences in the incidence and severity of side effects after the second or the first dose of the vaccine.

Side Effect	Incidence of Side Effects: Risk Ratio (95% CI)	Incidence of Side Effects: Multivariate Logistic Regression, Coefficient (*p*-Value)	Severity of Side Effects: Univariate Cumulative Risk Models (*p*-Value)	Severity of Side Effects: Multivariate Cumulative Risk Models (*p*-Value)
Any side effect	1.04 (1.01–1.07)	NS	NS	NS
Localized reaction	0.98 (0.94–1.03)	2.469 (<0.001)	NS	NS
Fever	1.72 (1.46–2.02)	1.3 (<0.001)	NS	NS
Flu-like illness	1.67 (1.45–1.91)	0.979 (0.001)	NS	NS
Shortness of breath	0.95 (0.57–1.61)	4.491 (<0.001)	NS	NS
Skin rash	2.25 (1.4–3.62)	4.297 (<0.001)	0.05	NS
Tingling	1.31 (0.89–1.92)	3.096 (<0.001)	NS	NS
Swelling	2.03 (0.87–4.77)	NS	NS	NS
Generalized swelling	1.2 (0.61–2.34)	4.925 (<0.001)	NS	NS
Anaphylaxis	2.54 (0.72–8.98)	4.747 (0.012)	NS	NS
Fatigue or tiredness	1.4 (1.28–1.53)	0.868 (<0.001)	NS	NS
Other	1.05 (0.83–1.32)	2.104 (<0.001)	NS	NS
		Worse outcomes after the second COVID-19 vaccine dose		

## Data Availability

The data presented in this study are available on request from the corresponding author.

## Appendix A

**Table A1:** Definitions of the severity of side effects.

Severity	Definition
**Minimal**	Negligible impact
**Mild**	No treatment needed
**Moderate**	Needed treatment or advice from healthcare professional outside the hospital
**Severe**	Needed hospital care

**Table A2:** Baseline characteristics of the study participants. Continuous variables are presented as medians (IQR) and categorical as *n* (%). Between group differences were anticipated and explained by the incidence of COVID-19 in different subgroups. Characteristically, a higher incidence of a prior COVID-19 infection was observed among frontline workers, health professionals and among British people (a very high incidence of COVID-19 was documented in the UK).

Characteristics	Participants with a Prior COVID-19 Infection (*n* = 532)	Participants with No Known Prior COVID-19 Infection (*n* = 1470)	Missing Data	Between Group Differences (*p*-Value)
**Gender (Female)**	393 (73.9%)	1051 (71.5%)	0.7%	NS
**Age ≥ 60 (%) ***	56 (10.5%)	202 (13.7%)	0.5%	NS
**Weight (kg)**	75 (64–88)	74 (64–85)	4.0%	NS
**Height (cm)**	168 (163–173)	168 (162–175)	2.2%	NS
**Country**			0.6%	<0.001
Europe				
UK	472 (88.7%)	1100 (74.8%)		
Greece	38 (7.1%)	294 (20%)		
Other European countries	10 (1.9%)	30 (2.0%)		
Americas	5 (0.9%)	17 (1.2%)		
Asia	5 (0.9%)	17 (1.2%)		
Africa	0 (0%)	1 (0.1%)		
**Ethnicity**			1.8%	NS
White	464 (87.2%)	1303 (88.6%)		
Asian	35 (6.6%)	63 (4.3%)		
Arab	21 (3.9%)	45 (3.1%)		
Other	7 (1.3%)	28 (1.9%)		
**Role**			3.2%	<0.001
Doctor	140 (26.3%)	486 (33.1%)		
Nurse	125 (23.5%)	188 (12.8%)		
Other health professional	161 (30.3%)	382 (26.0%)		
Not a health professional	105 (19.7%)	401 (27.8%)		
**Frontline workers**	372 (69.9%)	795 (54.1%)	0.6%	<0.001
**COVID-19 prior to vaccination**			0%	
Laboratory confirmed exposure	366 (68.8%)	NA		
Consistent symptoms, not tested	166 (31.2%)	NA		
No known exposure	NA	1470 (100%)		
**Vaccine type**			0.5%	NS
Pfizer	443 (83.3%)	1230 (83.7%)		
Oxford AstraZeneca	80 (15.0%)	202 (13.7%)		
Other	4 (0.8%)	20 (1.4%)		
Unknown	2 (0.4%)	3 (0.2%)		
**Vaccine preconception**			0.8%	NS
Positive	343 (64.5%)	1027 (69.9%)		
Neutral	76 (14.3%)	174 (11.8%)		
Negative	110 (20.7%)	259 (17.6%)		
**Second vaccine dose received**	114 (21.4%)	411 (28.0%)	0%	0.004
**Past medical history**			7.7%	
Chronic cardiac disease	9 (1.7%)	25 (1.7%)		NS
Chronic respiratory disease	74 (13.9%)	171 (11.6%)		NS
Chronic kidney disease	4 (0.8%)	9 (0.6%)		NS
Chronic liver disease	1 (0.2%)	6 (0.4%)		NS
Chronic neurological disease	8 (1.5%)	17 (1.2%)		NS
Active cancer	1 (0.2%)	9 (0.6%)		NS
Asplenia	1 (0.2%)	4 (0.3%)		NS
Allergy	56 (10.5%)	134 (9.1%)		NS
Diabetes	17 (3.2%)	49 (3.3%)		NS
Hay fever, eczema	114 (21.4%)	251 (17.1%)		0.04
Immunosuppression	14 (2.6%)	49 (33.3%)		NS
Transplantation history	0 (0%)	0 (0%)		NS
None	282 (53.0%)	825 (56.1%)		NS

* Participants with a prior COVID-19 exposure were younger compared with those without a prior exposure. See Figure A1.

**Table A3:** Differences in the incidence and severity of side effects after the first dose of a COVID-19 vaccine among participants who had or did not have a prior self-reported COVID-19 infection. Sensitivity analysis only included participants with a prior COVID-19 infection confirmed with a consistent PCR or antibody test (*n* = 366) versus those without any suspicion of a prior COVID-19 infection (*n* = 1470).

Side Effect	Incidence of Side Effects: Risk Ratio (95% CI)	Incidence of Side Effects: Multivariate Logistic Regression, Coefficient (*p*-Value)	Severity of Side Effects: Univariate Cumulative Risk Models (*p*-Value)	Severity of Side Effects: Multivariate Cumulative Risk Models (*p*-Value)
Any side effect	1.09 (1.05–1.12)	0.581 (0.015)	<0.001	0.004
Localized reaction	1.11 (1.06–1.16)	0.411 (0.019)	0.002	NS
Fever	2.45 (2.01–3)	0.902 (<0.001)	NS	NS
Flu-like illness	1.92 (1.61–2.29)	0.691 (<0.001)	NS	NS
Shortness of breath	2.06 (1.22–3.49)	0.564 (0.043)	NS	NS
Skin rash	1.38 (0.7–2.71)	NS	NS	NS
Tingling	1.22 (0.75–1.98)	NS	NS	NS
Swelling	0.73 (0.16–3.28)	NS	NS	NS
Generalized swelling	1.72 (0.8–3.73)	NS	NS	NS
Anaphylaxis	0.8 (0.09–6.85)	NS	NS	NS
Fatigue or tiredness	1.39 (1.24–1.56)	0.459 (<0.001)	<0.001	0.002
Other	1.45 (1.12–1.87)	0.288 (0.069)	NS	NS
		Worse outcomes associated with a prior COVID-19 infection		

**Table A4:** Differences in the incidence and severity of side effects among different ethnicities (white or other).

Side Effect	Incidence of Side Effects: Risk Ratio (95% CI)	Incidence of Side Effects: Multivariate Logistic Regression, Coefficient (*p*-Value)	Severity of Side Effects: Univariate Cumulative Risk Models (*p*-Value)	Severity of Side Effects: Multivariate Cumulative Risk Models (*p*-Value)
Any side effect	1.05 (0.99–1.11)	NS	NS	NS
Localized reaction	1.04 (0.97–1.12)	NS	NS	NS
Fever	0.62 (0.48–0.79)	–0.546 (0.003)	NS	NS
Flu-like illness	0.78 (0.62–0.97)	NS	NS	NS
Shortness of breath	1.16 (0.54–2.5)	NS	NS	NS
Skin rash	0.7 (0.32–1.56)	NS	NS	NS
Tingling	1.69 (0.79–3.61)	NS	NS	NS
Swelling	0.86 (0.2–3.81)	NS	NS	NS
Generalized swelling	0.64 (0.27–1.53)	NS	NS	NS
Anaphylaxis	0.66 (0.08–5.67)	NS	NS	NS
Fatigue or tiredness	0.88 (0.76–1.02)	NS	NS	NS
Other	1.38 (0.94–2.03)	0.446 (0.049)	NS	NS
		Worse outcomes: non-white ethnicity		
		Worse outcomes: white ethnicity		

**Table A5:** Differences in the incidence and severity of side effects among people with a different preconception toward the vaccine prior to vaccination and those who were keen to receive the vaccine versus those who were concerned about receiving the vaccine.

Side Effect	Incidence of Side Effects: Risk Ratio (95% CI)	Incidence of Side Effects: Multivariate Logistic Regression, Coefficient (*p*-Value)	Severity of Side Effects: Univariate Cumulative Risk Models (*p*-Value)	Severity of Side Effects: Multivariate Cumulative Risk Models (*p*-Value)
Any side effect	1.01 (0.97–1.06)	NS	<0.001	0.025
Localized reaction	0.99 (0.93–1.05)	NS	0.002	NS
Fever	1.19 (0.93–1.53)	NS	0.009	NS
Flu-like illness	1.07 (0.86–1.34)	NS	<0.001	NS
Shortness of breath	1.73 (1.00–2.98)	–0.085 (0.03)	NS	NS
Skin rash	1.25 (0.59–2.65)	NS	NS	NS
Tingling	2.23 (1.45–3.42)	–0.114 (0.001)	NS	NS
Swelling	0.4 (0.05–3.03)	NS	NS	NS
Generalized swelling	0.72 (0.26–2.04)	NS	NS	NS
Anaphylaxis	NA	NS	NS	NS
Fatigue or tiredness	1.17 (1.03–1.34)	NS	0.009	NS
Other	1.26 (0.96–1.66)	–0.043 (0.045)	NS	NS
		Worse outcomes: concerned		
